# A mixed-approach to investigate what motivates Belgian students to study medicine

**DOI:** 10.15694/mep.2020.000204.1

**Published:** 2020-09-22

**Authors:** Hélène Givron, Line Fischer, Martin Desseilles

**Affiliations:** 1 Faculty of Medicine; 2 Education and Technology Department

**Keywords:** medical education, curriculum, undergraduate medical students, motivations, choice of professional orientation.

## Abstract

This article was migrated. The article was marked as recommended.

Aims - Our main objective was to explore the motivations that led our third year Belgian students to study medicine.

Method - We used a mixed method as we collected both quantitative and qualitative measures at the same time through online surveys. Chi-Square tests were used to examine differences in motivation between gender of the participants. A thematic content analysis was then conducted on the open-ended question using a qualitative approach.

Results and conclusions - The final sample consists of 243 third-year students (87 men and 156 women). Chi-square analyses revealed gender differences in motivations to study medicine. The motivation most often reported by our students in both qualitative and quantitative analyses is “altruistic motivation”. The qualitative analysis identified, within this category, sometimes unrealistic beliefs about the practice of medicine, leading to the conclusion that it is necessary to put medical students in contact with the reality of the field as soon as possible. Furthermore, the lack of focus on the relationship with the patient within the formal curriculum and subsequent medical practice could be the cause of a form of disillusionment among these students. We believe that more lessons on relational aspects should be offered to them.

Perspectives - Finally, in addition to identifying and classifying the motivations present among medical students, we believe that some perspectives are the analysis of the interactions between these motivations in 1) the determination of the choice of study and 2) the attitudes and behaviours that will result from it.

## Introduction

Current physicians are criticized for a lack of empathic and communication skills ( Hojat et al., 2004; Bombeke et al., 2010; Neumann et al., 2011; Rosenbaum, 2017) but are applauded as heroes for their tireless involvement in the global health crisis of SARS-COVID-19. But what really motivates young people to study medicine? Is it precisely the desire to be in a relationship, to help others or do these studies select students seeking power and a certain social and financial status? The initial motivation to study medicine seems to be very interesting to explore because it may define future relational behaviours, impact the student’s professional development, his prioritization of tasks, as well as the time he will give to the establishment of a quality relationship with his patient ( Jeammet, Reynaud and Consoli, 1980).

According to the self-determination theory ( Deci et al., 1991), individuals differ in their level and type of motivation. Some studies have focused on the development of questionnaires measuring the level of the motivation that drive students to enter in medical studies (for example “how much would you like to study medicine, even if you have to spend a lot of time on topics that later turn out to be a waste of time”) ( Nieuwhof et al., 2004). Others were interested in the type of motivation, typically: intrinsic (i.e., practice an activity because of the interest and pleasure in doing it) vs. extrinsic motivation (i.e., practising an activity not because of the pleasure it gives, but for external, instrumental reasons) that pushed the students to carry out these studies ( Sobral, 2004; Wouters et al., 2017). Focusing on exploratory research on the content of motivations behind the choice to study medicine, Vaglum et al. ( Vaglum, Wiers-Jenssen and Ekeberg, 1999) summarized that there are 3 main categories of motivations influencing the choice to study medicine: “people oriented”, “natural science oriented motives” and “status/security oriented”. Although motivational factors may slightly vary from culture to culture, the results of this study carried out in 1999 have been continuously confirmed since then. More specifically, it is the « person oriented » motivation also called « altruism » that appears to be the primary motivation, most often cited by the students surveyed ( Hyppölä et al., 1998; Girasek et al., 2011; Korkmaz and Şenol, 2013; Sulong et al., 2014; Heikkilä et al., 2015; Halari et al., 2016; Wouters et al., 2017; Afzal et al., 2019). The second most reported motive is « interest in science » ( McManus, Livingston and Katona, 2006; Johansson and Hamberg, 2007; Korkmaz and Şenol, 2013; Sulong et al., 2014; Wouters et al., 2017). Status/security oriented factors have been identified in some studies too. In Ireland in more than a third of students identified « financial gain » as their one major motive ( Sulong et al., 2014); in Hungary, it was reported as 2 ^nd^ motive after humanitarian aspect ( Girasek et al., 2011), as well as in Finland, where it was reported as the second motive after « interactions with humans » ( Heikkilä et al., 2015). « Parental wish » is also often reported ( Seetharaman and Logaraj, 2012; Tiwari et al., 2017; Wouters et al., 2017). For example, in the study conducted in Pakistan by Afzal et al., 67% of students indicated it as one of the 4 most important reasons for entering medical school ( Afzal et al., 2019). Finally, “social status” was also reported as an important motivation by students ( Seetharaman and Logaraj, 2012; Wouters et al., 2017).

This literature provides a general picture of trends of motivations among students but they do not tell us about why students responded as they did and the deeper intentions that governed this orientation’ choice. Moreover, these motivations have been little investigated in our country. According to our review of the literature, only Boudrenghien et al. have recently investigated the motivations of Belgian students to study medicine but these researchers only used a quantitative approach ( Boudrenghien et al., 2015). Moreover, the literature on motivations in medical pedagogy frequently addresses this issue in the selection process for medical students. Our research group is interested in motivations as part of investigations into the determinants of communicative and empathic behaviours of medical students.

### Research question

Our main objective was to explore the motivations that led our third year Belgian students to study medicine. As a first step, we wanted to verify whether the categories generally found in the literature were present in our sample (quantitative approach). Secondly, we wanted to explore more broadly, through an inductive approach, whether other aspects appeared in the spontaneous responses of our students (qualitative approach).

## Methods

To reach our goal, as reported, we collected both quantitative and qualitative measures at the same time (i.e., simultaneous collection of both types of data ( Creswell, 1999)) using online surveys methodology. The methodological approach adopted here is therefore similar to a “mixed method”, defined by Creswell as a research design that combines both qualitative and quantitative methods for data collection and analysis in a single study ( Creswell, 1999). The mixed method, allowing a broader and deeper understanding of a phenomenon seemed to be the most adequate approach to reach our goal. More specifically, in this research, more attention will be paid to qualitative material to understand precisely the motivations for the medical orientation of the targeted sample, while quantitative analyses will be more introductory. However, both types of data will be integrated into the final discussion.

### Data collection

We collected data about age, gender and motivations. For the motivations, students were first asked to answer to an open-ended question « What were your reasons to study medicine? ». Second, they were asked to tick among 18 items those they believed were behind their decision to study medicine. These items were identified in the literature on motivations to study medicine ( Missenard, 1976; Rolfe, Ringland and Pearson, 2004). We focused on the type, not the level of motivation of our medical students. However, we do not rely on the “intrinsic” vs. “extrinsic” distinction ( Deci and Ryan, 2000), which we consider a priori to be too dichotomous. The options presented in random order to students are listed below in the Table 1.

The qualitative data collection (open-ended question) was carried out before the quantitative data collection in order not to bias the students’ responses.

To sum up, in addition to collecting data of different types, this questionnaire first allowed an exploratory investigation of students’ motivations, and then to study their position on the motivations previously identified in the literature on motivations for studying medicine (20 items). This approach to data collection was consistent with the goals of this study, as this study explored whether the motivations identified by the authors of this field were present in the target sample, in what proportion, and whether any other trends emerged from the data. The demographic variables allowed for a preliminary quantitative analysis of the data in order to analyze the relationship between gender and motivations in our sample. The choice to explore motivations by gender is supported by the literature ( Millan et al., 2005; McHugh et al., 2011) which shows that there are differences in the motivations for entering medical school between men and women.

### Procedure

At the end of their third year of study (in Belgium, the basic university curriculum in medicine -before any specialization- is made of 6 years), a self-questionnaire is proposed, on a voluntary and unpaid basis, to all third-year medical students enrolled at the University of Namur before and after their first internship, in order to evaluate its impact. We benefitted from this data collection by inserting our questions on motivations into their pre-internship questionnaire. Information about the questionnaire was given to students at the end of one of their lessons. The link to the questionnaire was open access on the “virtual laboratory” (website of the psychology department offering different surveys to students). The data were collected from June to July 2017. Before completing the questionnaire, students were asked to read and accept the informed consent form to participate freely in this study. This study was approved by the local Ethics Committee of “Cliniques Universitaires UCL Mont-Godinne” and conducted in accordance with the Declaration of Helsinki.

### Data Analysis

• Quantitative analysis

Quantitative analysis were performed with SPSS (Statistical Package for the Social Sciences), version 24 (IBM). Frequencies and percentages are illustrated in Table 1. Chi-Square tests were used to examine differences between gender of the participants (male VS female). The application conditions were found (independent observations and mutually exclusive categories of variables). For one item (family pressure), the minimum expected count was less than 5 and therefore the test could not be applied. A p-value less than 0.05 was considered statistically significant.

• Qualitative analysis

A thematic content analysis was then conducted on the open-ended question using a qualitative approach ( Bardin, 1977; Miles and Huberman, 1994). The purpose of this qualitative analysis was to understand deeper the motivations of medical students to study medicine. It was conducted manually in light of the limited amount of data (243 responses over 17 pages). Firstly, the students’ responses were read for the first time by the researcher in order to retain only interpretable data (i.e., suppression of answers that were illegible or not related to the question asked). Next, the responses of the 243 students were copied into a working document, which served as the basis for the following steps. The third step was carried out by two independent researchers. Indeed, two researchers carried out the coding of the entire material independently. They labelled the motivations within the verbatims using low-level abstraction codes until they gradually created more abstract codes to arrive at eight categories, which made it possible to draw up a final coding grid. Within this grid, the main categories and sub-categories were organized. For example, the category “pro-social motivation” was separated into two sub-categories: social motivation at a micro level (assistance to relatives or patients) and at a macro level (assistance to society, citizen involvement). The categories created are exhaustive (they cover the entire material) and exclusive (a motivation can only be coded in one category). Following this operation, the two researchers compared their coding grid. Although the analyses were close, some disagreements were discussed which helped to refine the analysis and increase its reliability. Following this step, the data from the open-ended question were integrated into a final coding grid where the occurrences of each motivation were counted (see Appendix 1). This quantification of the qualitative material gave an idea of the frequency of certain responses within the student sample. After counting the occurrences, different motivations emerged as priorities in the data. We will give them a special place in the presentation and analysis of the results. Finally, following the development of this coding grid using an inductive approach, the scientific literature highlighting the motivations of students to choose a medical course was consulted in order to put the data into perspective with existing theories and to allow triangulation.

### Participants

The study was conducted in June 2017 with 254 students. The final sample consists of 243 third-year students (87 men and 156 women). We thus have a participation rate of 95.67%. The average age of the responding students is 21.9 years (SD = 2.15). It should be noted that until September 2017, any student (i.e. the participants in this study) who obtained the higher education diploma could enrol in the first year of medicine without first having to pass a selection process at the start of their studies.

## Results/Analysis

The results of the quantitative survey will be presented before those of the qualitative analysis. The quantitative analysis will allow us to verify that the motivations usually found in other studies are also present in our students. The qualitative analysis will then allow us to go one step further to enhance our understanding of the preliminary results.

### Quantitative results

**Figure 1.**
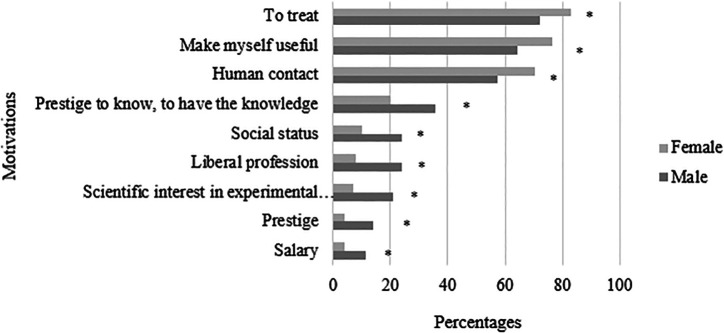
Illustration of significant percentage differences in reported motivations by gender. Note. *p < 0.05

The descriptive data (frequencies and percentages of items selected by students) are summarized in the Table 1. They are presented in descending order (from the most ticked to the least ticked). Chi-square analyses were then conducted to explore whether, as in the literature, we observe gender differences in motivations to study medicine. As shown in Figure 1, significant X² were found for these items : to treat (X² = 4.08, p < 0.05); make myself useful (X² = 3.93, p < 0.05) ; human contact (X² = 4.22, p < 0.05) ; scientific interest in experimental sciences (X² = 9.89, p < 0.05); prestige to know, to have the knowledge (X² = 7.3, p < 0.05) ; social status (X² = 8.34, p < 0.05) ; liberal profession (X² = 12.87, p < 0.05) ; prestige (X² = 11.45, p < 0.05) ; salary (X² = 5.31, p < 0.05).

**Table 1.** Frequencies and percentages of motivations of females and males to study medicine.

Items	Male n (%)	Female n (%)	X²	p*	Total N (%)
To treat	63 (72%)	130 (83%)	4.08	0.044 *	193 (79.4%)
Make myself useful	56 (64.4%)	119 (76.3%)	3.93	0.047 *	175 (72%)
Understanding how the body works	64 (74%)	104 (67%)	1.25	NS	168 (69%)
Human contact	50 (57.5%)	110 (70.5%)	4.22	0.040 *	160 (65.8%)
To cure	51 (59%)	107 (69%)	2.44	NS	158 (65%)
Scientific interest	47 (54%)	69 (44%)	2.15	NS	116 (47.7%)
Scientific interest in humanities and social sciences	30 (34.5%)	51 (33%)	0.08	NS	81 (33.3%)
Scientific interest in biology	32 (37%)	40 (26%)	3.33	NS	62 (29.6%)
Responsibility	28 (32%)	40 (26%)	1.19	NS	68 (28%)
Prestige to know, to have the knowledge	31 (35.6%)	31 (20%)	7.3	0.007 *	62 (25.5%)
Social status	21 (24.1%)	16 (10%)	8.34	0.004 *	37 (15%)
Theoretical scientific interest	16 (18.4%)	17 (11%)	2.67	NS	33 (13.6%)
Liberal profession	21 (24.1%)	12 (8%)	12.87	0.000 *	33 (13.6%)
Personal safety	16 (18.4%)	16 (10.3%)	3.23	NS	32 (13%)
Scientific interest in experimental sciences	18 (21%)	11 (7%)	9.89	0.002 *	29 (12%)
Prestige	12 (14%)	4 (3%)	11.45	0.001 *	16 (6.6%)
Salary	10 (11.5%)	6 (4%)	5.31	0.021 *	16 (6.6%)
Family pressure	6 (7%)	0 (0%)	11.03	/	6 (2.5%)

* Statistically significant at p<0.05 ; / minimum expected count < 5

### Qualitative results

The thematic content analysis carried out on the data led to the development of a coding grid common to both researchers, which categorized the 8 different types of motivations reported by the students. This section therefore aims to present the content of this coding grid as well as the organization of the categories of motivations that emerged from the data (grid presented in the Appendix 1). It is important to note first that 528 motivations were coded by the researchers in the responses of the 243 students. Each student invoked, on average, not just one type of motivation, but rather two.

Each category and sub-category will be explained and illustrated through different verbatims highlighting the motivation in the students’ discourse (alias names will be used to preserve the students’ anonymity). As announced, the frequency of appearance of each of the motivations will be provided. The names of the different categories have been taken from the students’ vocabulary as much as possible (e.g. “interest in science”) to stay as close as possible to the data. However, some have been restated by the researchers (e.g. “altruistic motivation”). The different motivations are reported below by frequency order, as they appear in the annexed grid.

1. The motivation most frequently found in the data is **altruistic motivation** to enter medical school. This was reported by 94% of the students in the sample. In this category, the student said that this job would allow him to be in contact with patients, to care, to help others and even to “save” lives. Two sub-categories were noted: a motivation to be useful to patients or their relatives (at a “micro” level) was differentiated from a more “macro” level motivation to be useful to society, to the world (for example, by participating in humanitarian missions). The first is reported by 75% of the students, as Léa explains “I have always loved listening to people, helping them and having compassion for them”. The second sub-category is less frequently reported (only 19%) of the students refer to it)
2. A second type of motivation concerns the **student’s interest in the scientific field**, mentioned by 76% of students. Within this category, a first sub-category includes students who cite an attraction for the medical curriculum (17%) as it is organised at the university (internships, diversity of courses...) and for its difficulty or the challenge that its success represents. Moreover, the second sub-category is attraction in scientific courses (and precisely, the understanding of the functioning of the human body) and the intellectual approaches and postures they require (investigations, hypotheses, research, evolution of knowledge...). About one in two students mentions this motivation for science (83%). Noah explains, with reference to this second sub-category, “I wanted to move into a scientific field that requires a constant intellectual and critical approach, both to perfect some of my knowledge and to infirm others[...]”.
3. The third category of motivation relates to **the attractiveness of the medical profession and its characteristics** (liberal profession, diversity of opportunities and tasks, autonomy of decision-making...) and is mentioned by 25% of the students surveyed. Johan indicates, for example, “that these studies offer him a huge number of career choices”.
4. A fourth type of motivation to start medical studies is **vocation**. This is, for us, a category in its own right where the student does not detail the reasons for his choice of orientation but makes explicit, like Thomas, “that he has always wanted to do these studies” or like Gregory, “that he has always known that he wanted to become a physician”. 18% of the students invoke a choice of orientation based on this principle.
5. The fifth category (called “ **social influence**”) concerns the 8% of students who explain that they made this choice of study thanks to someone close to them, who served as a “role model”. Within this category, we have differentiated a first sub-category when the influence comes from a family member practising a health profession (7%) and a second sub-category when this influence is not exerted by a close relative but by a doctor with whom the student has been in contact (only 1%). Arnaud explains “Although I don’t like to equate my decision with what some people might say - he went to medical school, like his father - my father being a general practitioner, I have had the opportunity to observe him in his practice since I was a young boy”.
6. The **student’s confrontation with the disease** forms a sixth category of motivation (5%), also divided into two different subcategories depending on whether the student has experienced the disease himself (2%) or a relative (3%). Rita recalls, “My grandparents died when I was young, and even then I wanted to do something for them, to find a solution to their problems”. This excerpt has been coded in this second sub-category since this is her grandparents’ illness.
7. The penultimate motivation concerns a choice for these studies guided **by social, financial or status recognition.** For example, Max reports that “the financial aspect is not negligible” in his choice. Only 4% of the students expressed this idea in their response. It should be noted that the status and financial gain have been grouped in this same category given the small number of students referring to it.
8. Finally, the last motivation very rarely found in our data (2%) was named “ **the default choice or lack of motivation**” by the researchers and refers to students who do not express specific motivations for these studies or the resulting profession but who seem to make this choice by spite or lack of attraction towards other orientations. Louise notes, for example, “at first I started studying medicine because I didn’t know what to do..."

## Discussion

First, the quantitative analysis allowed us to identify that the two motivations most often reported by students for entering medical school are « to treat » and « make myself useful », followed by a more scientific motivation: « understand how the body works ». The 3 items least often selected are « prestige », « salary » and « familial pressure ». These results are consistent with those found in the literature indicating that the first motivation most often reported by students is altruistic, followed by scientific motivations and finally socio-economic/status-related motivations ( Hyppölä et al., 1998; Vaglum, Wiers-Jenssen and Ekeberg, 1999; McManus, Livingston and Katona, 2006; Johansson and Hamberg, 2007; Girasek et al., 2011; Korkmaz and Şenol, 2013; Sulong et al., 2014; Heikkilä et al., 2015; Halari et al., 2016; Wouters et al., 2017; Afzal et al., 2019). Finally, the significant differences found in the quantitative analysis between the motives reported by men and women supported those found in the literature too ( Millan et al., 2005; McHugh et al., 2011). As expected, we find more motivation for aspects related to the “social status”, “prestige”, “salary” associated with the profession among men and more motivation for “human contact” among women ( Korkmaz and Şenol, 2013; Heikkilä et al., 2015; Halari et al., 2016).

Second, at the qualitative level, the first type of motivation, grouped under the category “altruistic motivation”, was most often cited by students as motive to study medicine. This result confirms the one found for decades now ( Hyppölä et al., 1998; Girasek et al., 2011; Korkmaz and Şenol, 2013; Sulong et al., 2014; Heikkilä et al., 2015; Halari et al., 2016; Wouters et al., 2017; Afzal et al., 2019). Like other authors ( McManus, Livingston and Katona, 2006; Johansson and Hamberg, 2007; Korkmaz and Şenol, 2013; Sulong et al., 2014; Wouters et al., 2017), the second most frequently cited verbatims were listed under the category “interest in the scientific field”. Next, the fact that the 3rd and 4th motivations were “attractiveness of the medical profession and its characteristics” and “vocation” is rather a good thing, since researchers have shown that these motivations are associated with significant subsequent satisfaction in practice ( Heikkilä et al., 2015). The category “vocation” has been found in other studies ( Heikkilä et al., 2015; Wouters et al., 2017), and also seems to fit Woodward’s « child’s dream » category ( Woodward et al., 2017). The 5 ^th^ category « social influence of a family member practicing medicine or a related profession » includes reasons also found in the qualitative study of Woodward et al.: « inspired by role models » and « influence of family members ». On the other hand, unlike other studies reporting “family pressure” ( Seetharaman and Logaraj, 2012; Pruthi et al., 2013; Tiwari et al., 2017; Wouters et al., 2017; Afzal et al., 2019), no student reported qualitatively having experienced family pressure to study medicine. However, this item was selected six times in the quantitative section. The 6 ^th^ category « experience of the disease » has also been found by other authors ( McHarg, Mattick and Knight, 2007; Pagnin et al., 2013). Indeed, Pagnin and De Queiroz ( Pagnin et al., 2013) identified that medical students who expressed this motivation scored higher on the “emotional exhaustion” component of burnout that students with other motivations. Conversely, altruistic motivation has been proposed as a protective factor against burnout and depersonalisation in healthcare ( Győrffy, Birkás and Sándor, 2016). Probably for social desirability reasons, the 7 ^th^ category “social, financial and status recognition” that includes all the socio-economic attractions of the medical profession is less frequently mentioned by our students. Finally, the 8 ^th^ category “the default choice” seems, like the 4 ^th^ category (vocation), of a different nature, as it could conceal other motivations, of which the student is not aware or which he does not make explicit. As Amin et al. point out in their article, it is surprising to see that no student reports an interest in working with peers or in teams, in the workplace ( Amin et al., 2009) since teamwork and multidisciplinary are a major aspect of medical practice. This highlights how representations of the medical profession include or exclude certain aspects of the profession.

By integrating the results emerging from the qualitative and quantitative analyses, we notice, firstly, that the motivation most often reported by our students in both types of data is altruistic. The items « to treat » and « to make myself useful » (most frequently checked at the quantitative level) seem to us to relate to the first category found at the qualitative level that we have named « altruistic motivation ». However, the qualitative analysis allowed us to see a heterogeneity of responses within this category that was not possible with the quantitative analysis alone. Indeed, this altruistic motivation may be associated either (1) at a “micro” level, with a desire to be useful to one’s family, one’s patients; or (2) at a more “macro” level, with a desire to be useful to society, in particular by targeting the most deprived. This suggests that the choice of orientation may be linked to personal values (here, helping others or make a contribution). Another clarification emerged from reading the verbatims (semantically speaking): the existence of a range of nuances in students’ beliefs about their ability to help patients. Some students say they want to help, treat and if possible cure patients. Others claimed to study medicine to save lives, some even making a comparison with the profession of “superhero” (we can guess the influence of the medical films and series that young adults are fond of). This raises questions about the sometimes unrealistic beliefs that drive students to enter medical school. It’s worth noting that students we interviewed are in pre-clinical years; so they have had no internship/contact with patients as part of their formal curriculum. This lack of concrete experience may therefore provide them with an idealized vision of their future profession. This position may have some advantage in terms of commitment and perseverance in studies. Indeed, these unrealistic beliefs may have the benefit to keep the student in a certain denial and to avoid dissonance thanks to a positive and control bias on the profession allowing them to remain motivated in these difficult studies. However, this vision carries the risk of a “shock” in the face of reality during the first internship. Students’ interactions with medical staff have been implicated in the decline of the idealism students present towards their future profession ( Morley et al., 2013). Some authors argue that if this “idealistic” motivation does not meet the practical experience, some students may feel disillusioned and may withdraw from medical education ( Girasek et al., 2011; Woodward et al., 2017). One study showed that one-third of medical students would never do these studies again ( Pruthi et al., 2013). This leads us to conclude that it may be important to bring students more quickly into contact with practice as part of their undergraduate curriculum, so that they can identify whether they really like it and what skills they will need to develop to find their place between the patient and the health care system. Moreover, in our sample, we observe only one student out of twenty living in a family medical environment who reports studying medicine to “save lives”; the others having used a more nuanced vocabulary (help, care). Thus, we make the assumption that a better knowledge of the environment may offer a more realistic vision of the profession to students. However, rather than trying to match students to the reality of current practice (where physicians are criticized for a lack of empathy and communication skills ( Hojat et al., 2004; Bombeke et al., 2010; Neumann et al., 2011; Rosenbaum, 2017)), perhaps the focus should also be on better aligning medical education and practice with the expectations of students entering medical school. Indeed, it is questionable to think that the primary motivation of students entering medicine is pro-social, yet neither their future profession nor the curriculum seems to focus primarily on this aspect. The practice, but also the content of the studies can therefore be the cause of a form of disillusionment among medical students. It is known that undergraduate medical students appreciate and demand more practical courses that train their relational and communication skills ( Givron and Desseilles, 2019).

Still concerning the undergraduate curriculum of medical students, it seems to us that discussing motivations with them, for example in psychology courses, can be an interesting introspective exercise. Not with the aim of changing them, but to make them more flexible and to increase their self-knowledge so that they can take their motivations into account in their future relationships with their patients and in their choice of work environment or medical speciality. One can imagine, for instance, that our student who answered “I can’t watch people die without trying to do something for them” will be in great difficulty when faced with the first loss of one of her/his patients. The other student who answered “I want to save lives” might not be able to see her/his need for repair defeated. However, Deci et al. argue that persistent deprivation of a need has a cost for well-being and health ( Deci and Ryan, 2000). Thus, these situations will make these students particularly anxious and could lead them to take an excessively supportive stand towards the patient or conversely, excessive detachment leading to depersonalization.

As said above, studies have shown a loss of empathy among medical students as they progress through study and practice ( Hojat et al., 2004; Bombeke et al., 2010; Neumann et al., 2011). We have already discussed below how a gap between unrealistic beliefs and reality can impact students and it would be interesting to investigate to what extent this change in empathy is related to changes in beliefs about the medical profession, or even changes in reported motivations to practice medicine. Indeed, authors have also reported that when a need is not met, it can lead to the development of “need substitutes” or “compensatory motives” that do not really satisfy the thwarted basic needs but provide some collateral satisfaction. The authors give the following example: if a young people’s need for relatedness is substantially thwarted, he will compensate by attempting to gain sense by pursuing image-oriented goals, such as accumulating money. The problem is that the initial and important need has still not been met and the deleterious consequences mentioned above (on health and well-being) may therefore remain ( Deci and Ryan, 2000). It is questionable to what extent this phenomenon can occur in a physician whose initial motivation to be in contact with patients is not met.

Furthermore, we would like to insist on the fact that although similarities can be found in the students’ verbatim reports (categories of general motivations), the qualitative analysis showed us how varied the paths from which these motivations emerged could be (e.g., stemming from a desire to care for a sick parent, identification with a role model doctor, etc.). Motivation, by definition, remains an essentially personal matter. What counts in the end, beyond making a list of the type of motivations that can lead to a caring function, is how these motivations will interact with each other to determine (1) the decision to study medicine and (2) the use that will be made of these motivations while practicing the profession. Thus, we invite further research to focus on how motivations interact, how they evolve, and how much weight each has in determining career choice or future professional behaviours. With regard to the interaction between motivations, our qualitative analysis allows us to draw some suggestions. Indeed, it has allowed us to identify that if for some students, a single motivation is at the origin of their choice to study medicine, there are on average 2 motivations at the origin of this choice. In some cases, the student clearly indicated that the weight given to one category was higher than the others, but in many cases, 2 motivations were equal and it was precisely the synergy between these two motivations that had decided them: the fact that medicine made it possible to mix both the sciences and the human being. Finally, based on the expectancy-value model ( Eccles and Wigfield, 2002), which aims to present the determinants of commitment to a task, it can be postulated that some categories of motivations are more distal than others. Indeed, in this model, the authors consider that previous experiences and socialization are distal factors that will impact the choice to engage in a task, but in a more indirect way than other, more proximal factors, such as the subjective task-value or the expectations of success. While some authors (9) have begun to model the antecedents of motivations to study medicine and the links between them, further research in this direction seems promising.

In closing, different limitations of this study must be pointed out. The first limitation inherent in measuring students’ motivations to study medicine is social desirability bias. It is possible that the percentage of students who report to study medicine for the associated status or salary is underestimated. Second, our sample is composed of 3rd year medical students. Although these students are still in pre-clinical years, it is possible that having taken courses for two years may have led them to develop biases. Recall bias is however unlikely since studies have shown that the motives for choosing a career in medicine appear to be relatively stable during medical school ( Scott et al., 2012). Finally, it is possible that the fact that the researchers were also involved in teaching a few hours of communication skills training (CST) may have influenced student responses by increasing the social desirability bias for the human dimensions. However, in order to minimize this bias, this questionnaire was proposed to students 6 months after the CST and presented as part of a pre-internship questionnaire to analyze the effect of the internship.

## Conclusion

To conclude,the motivation most often reported by our students in both qualitative and quantitative analyses is “altruistic motivation”. As in other studies, we also found that male students reported significantly more motivation related to the status of the medical profession and women reported more altruistic motivation. Qualitative analyses have identified, within the altruistic motivation, sometimes unrealistic beliefs about the practice of medicine. A risk of disillusionment may occur among students who report “altruism” as their primary motivation, realizing that this dimension is not always emphasized, either during study or in practice. Offering clerkships at the beginning of medical school can allow students to be quickly put in contact with the reality of the field. Moreover, we believe that more lessons on relational aspects should be offered to them. Finally, in addition to identifying and classifying the motivations present among medical students, we believe that some perspectives are the analysis of the interactions between these motivations in 1) the determination of the choice of study and 2) the attitudes and behaviours that will result from it.

## Take Home Messages

• The most often reported motivation is altruistic motivation.
• Men report significantly more motivation related to the status of the medical profession and women report more altruistic motivation.
• Offering clerkships early in medical school may help to quickly confront unrealistic beliefs held by some students.
• A risk of disillusionment may occur among students who report “altruism” as their primary motivation, realizing that the focus is not always placed on this dimension either during study or in practice.
• Research on the motivations of medical students should address the interaction of motivations in determining study choice and in the resulting attitudes and behaviours.

## Notes On Contributors

**HELENE GIVRON** is MSc in psychology, currently working as an Assistant-Doctorant in biomedical sciences (in 4 ^th^ year). She is supervising communication skills training at Namur University (Department of Psychology). She leads studies on medical students’ attitudes towards communicative skills, their level of empathy and wellbeing. ORCID number: https://orcid.org/0000-0002-7213-5460

**LINE FISCHER** is a clinical psychologist (MSc) and currently works as a pedagogical advisor for freshmen university students. She conducts researches in the field of clinical and educational psychology within the department “Education and technology” of the University of Namur. Her doctoral thesis work focuses on emotional regulation in learning. ORCID number: https://orcid.org/0000-0002-2466-3162

**MARTIN DESSEILLES** (MD, MSc, PhD) is a psychiatrist and psychotherapist, Professor at Namur University (Belgium) where he is the Director of the Department of Psychology. He divides his time between clinical practice, teaching and research. He is involved in training pharmacist, medical students and trainees in psychiatry. ORCID number: https://orcid.org/0000-0003-4748-3401

## Appendices

**Appendix 1.** Coding grid: classification of motivations to study medicine

Kind of motivation	Sub-categories	Explanations of the category	Illustrations of the category	Frequency order
**1) ** **Altruistic motivation** - To help - To care - To save - To be in link/in contact	Feeling useful for relatives, patients (micro level)	The student claims that this job allows her/him to be in human contact, to help others, to improve the quality of life of family members or patients.	- I have always loved listening to people, helping them and having compassion for them - I wanted a job with contacts	**182**
	Feeling useful for society (macro level)	The student invokes that she/he wants to be useful to society, to the world (for example, by carrying out humanitarian missions).	- I wanted to feel useful to society in a more invested way than a common citizen	**46**
**2) Interest in the scientific field**	Interest in the course or curriculum	The student invokes an attraction for the medical curriculum organized at the university (internships, diversity of courses...) and for its difficulty, its requirement, the challenge that the success of these studies represents.	- In addition, I found the studies very interesting and exciting - It was said to be difficult	**26**
	Interest in scientific field	The student invokes an attraction for:- science courses (or more specifically the functioning of the human body)-the process of investigation, research, intellectual stimulation and the posture necessary for the development of knowledge in scientific disciplines.	- [...] because I’m passionate about the human body and the way it works (or malfunctions) - I wanted to move into a scientific field that requires a permanent intellectual and critical approach, both to perfect some of my knowledge and to infirm others and where knowledge is constantly advancing	**124**
**3) Attractiveness of the medical profession and its characteristics**	**/**	The student invokes characteristics of the profession that she/he considers attractive (except the social aspects): the diversity of career choices, the variety of tasks, the attraction towards technical aspects or for the medical and hospital environment, the autonomy in decision making, the self-employed status...	- I have always loved to learn new things, and medicine is such a vast subject that it can satisfy this passion - That these studies offer me a huge number of career choices	**61**
**4) Vocation**	**/**	The student invokes “that she/he has always wanted to do this” without explaining her/his motivations.	- I always wanted to study medicine - I always wanted to be a doctor	**43**
**5) ** **Social influence of a family member practicing medicine or a related profession**	Influence of a family member practicing a profession in healthcare	The student claims that she/he chose this profession because a member of her/his family works in healthcare.	- In addition, I was very interested in the medical field, my mother being a physiotherapist. So I was hesitating between pharmacy, medicine or biomedical sciences - Although I don’t like to equate my decision to what some people might say; “he went to medical school, like his father”. My father being a general practitioner, I have had the opportunity to observe him in his practice since I was a young boy	**16**
	Influence of a physician	The student mentions a physician she/he met who made her/him want to do these studies.	- [...] the people who have made a miraculous recovery thanks to the care of some great doctors - I’ve always enjoyed doctor’s visits as a child	**4**
**6) Experience of the disease**	Of the student	The student claims that she/he chose these studies because she/he was confronted with the disease herself/himself.	- In the past I have had serious health problems but today I am in complete remission	**5**
	Of a relative	The student claims that it was because someone close to him was confronted with the disease that he decided to undertake these studies.	- My grandparents died when I was young, and even then I wanted to do something for them, to find a solution to their problems	**8**
**7) Social, financial and status recognition**	**/**	The student claims that this choice of study gives rise to the recognition of relatives (admiration) or financial/status recognition, prestige, etc.	- It’s also a profession that arouses a lot of admiration from the people around us - The financial aspect is not negligible either	**9**
**8)The default choice/absence of motivation**	**/**	The student claims that she/he did not know what else to choose or that she/he was only interested in these studies.	- I couldn’t see myself doing anything else	**4**
