# Supplementary material for: A mixed-approach to investigate what motivates Belgian students to study medicine
Source: MedEdPublish (2016). 2020 Sep 22;9:204. [Version 1] doi: 10.15694/mep.2020.000204.1 (PMC10699379; doi:10.15694/mep.2020.000204.1)
Supplement: Peer review history

# Reviewer report: Reviewer response for version 1

**DOI:** 10.21956/mep.20111.r31011

**Published:** 2020-09-27

**Authors:** Shamaila ijaz

**Affiliations:** 1 Lisca

This review has been migrated. The reviewer awarded 5 stars out of 5 The paper's aim was to explore the motivations that led third year Belgian students to study medicine. The abstract was comprehensive with aim, method, results and conclusion.The introduction nicely defines five types of motivational factors of being a physician. The past literature is merged with present in a systematic manner. The motivation theories are relevantly described with the motives of physicians which contradict with general view about them having "lack of empathy and poor communication skills".The method section show the sampling and procedure of the research. The statistical analysis was used is chisquare. The sample consisted of 243 students (87 men and 156 women). The results showed altruistic motivation, research, the attractiveness of the medical profession and its characteristics, vocational, social influence, student's confrontation with the disease, by social and financial or status recognition.The discussion merged present literature with the previous literature and explained the results nicely. the qualitative part was very interesting. The suggestion of using psychology in initial level is a great idea.

Reviewer Expertise:

NA

No decision status is available

# Reviewer report: Reviewer response for version 1

**DOI:** 10.21956/mep.20111.r31013

**Published:** 2020-09-23

**Authors:** Mohamed Al-Eraky

**Affiliations:** 1 University of Dammam

This review has been migrated. The reviewer awarded 4 stars out of 5 Well done! I concur with Patricia that the methodology of this study is clear and can be easily replicated in other context for the same purpose. I also think that the points raised by Ravi would offer a comprehensive view on the context and the program of the medical school in the study. I would like to propose two extra points/studies. First a follow up study to ask the same students prior or after graduation, in case if they still have the same motives or their dreams have been transformed somehow. Also, authors might consider a complementary study to investigate the socio-economic status of medicine (as a profession), as perceived by other stakeholders in Belgium. How the Belgian society perceive doctors? This would shed light on the social contract and how it would contribute to choosing medicine as a profession for future generations. Thank you.

Reviewer Expertise:

NA

No decision status is available

# Reviewer report: Reviewer response for version 1

**DOI:** 10.21956/mep.20111.r31012

**Published:** 2020-09-23

**Authors:** P Ravi Shankar

**Affiliations:** 1 American International Medical University

This review has been migrated. The reviewer awarded 4 stars out of 5 This is an interesting manuscript on motivations to study medicine among students at a university in Belgium. The motivations have been organized by the authors into a systematic framework. My suggestions for further improvement are as follows:A description of the undergraduate medical program at the university will be helpful. Why did the authors select third year students for this study? How are students selected for the medical course? What are the tuition fees for the course and is there state subsidy for the fees? The authors mention that any student who has completed a high school diploma could enrol for the course? How many students are admitted to the medical program every year at the university and in Belgium? Family pressure to study medicine is not uncommon among Asian students and students of Asian descent. I believe the authors only collected information on gender in their study. The authors mention that the qualitative study was done first. How did the authors work out the themes and issues to be addressed during the qualitative study? Was a separate qualitative study conducted? Or was this data obtained from the quantitative study? What method was used? What do the authors mean by the first internship?

Reviewer Expertise:

NA

No decision status is available

# Reviewer report: Reviewer response for version 1

**DOI:** 10.21956/mep.20111.r31010

**Published:** 2020-09-22

**Authors:** PATRICIA CURY

**Affiliations:** 1 FACERES

This review has been migrated. The reviewer awarded 5 stars out of 5 This is an excellent paper about what motivates Belgian students to study medicine, congratulations! Methodology is described carefully and the discussion is very interesting! I would like to know more about social- economical background of this students. Are many difference between these students, as “very poor and rich” ? I suppose not, but this could interfere in the motivation. Another factor is the student´s religious formation and beliefs. When the authors say that “the choice of orientation may be linked to personal values”, religious beliefs could also interfere in their motivation. Do you have these data?

Reviewer Expertise:

NA

No decision status is available
